# Single-cell transcriptomics reveals the regulative roles of cancer associated fibroblasts in tumor immune microenvironment of recurrent osteosarcoma

**DOI:** 10.7150/thno.73714

**Published:** 2022-08-01

**Authors:** Xin Huang, Lutong Wang, Haoyu Guo, Weiyue Zhang, Zengwu Shao

**Affiliations:** 1 Department of Orthopaedics, Union Hospital, Tongji Medical College, Huazhong University of Science and Technology, Wuhan 430022, China.; 2 Department of Endocrinology, Union Hospital, Tongji Medical College, Huazhong University of Science and Technology, Wuhan 430022, China.

**Keywords:** Single-cell transcriptomics, Cancer associated fibroblasts, Tumor immune microenvironment, Osteosarcoma

## Abstract

**Rationale:** Osteosarcoma (OS) is the most common primary bone tumor with a poor prognosis, but the detailed mechanism is still unclear. A comprehensive investigation of tumor microenvironment (TME) of OS might help find effective anti-tumor strategies. Single-cell transcriptomics is a powerful new tool to explore TME. Therefore, this study is designed to investigate the TME and gene expression pattern of primary and recurrent OS at the single-cell level.

**Methods:** The single-cell RNA sequencing and bioinformatic analysis were conducted to investigate the cellular constitution of primary, recurrent, and lung metastatic OS lesions according to the datasets of GSE152048 and GSE162454. TIMER database was used to investigate the role of LOX in the prognosis of sarcoma. The functions of related cells and markers were further confirmed by *in vitro* and *in vivo* experiments.

**Results:** Cancer associated fibroblasts (CAFs) were found with a higher infiltrating level in recurrent OS, and were enriched in the epithelial-mesenchymal transition (EMT) pathway. CAFs showed remarkably increased expression of LOX, which might lead to EMT and poor prognosis of OS. Mechanically, LOX regulated the function of CAFs and macrophage polarization to remodel the tumor immune microenvironment. Moreover, LOX inhibitor could inhibit migration and promote apoptosis of OS both *in vitro* and *in vivo*.

**Conclusions:** This study revealed the heterogeneity of recurrent OS and highlighted an innovative mechanism that CAFs regulate EMT of OS via LOX. Targeting LOX of CAFs showed promising efficacy in remodeling TME and treating recurrent OS.

## Introduction

Osteosarcoma (OS) is one of the most common bone tumors, and mainly affects adolescents 1. In spite of substantial achievements in surgeries and chemotherapies, the prognosis of OS patients remains unsatisfactory 2, 3. Recurrent OS is one major clinical challenge and recurrent OS patients have a poor prognosis 4. Accordingly, it is urgent to explore the mechanisms of OS progression and enhance the understanding of recurrent OS thoroughly.

Tumor microenvironment (TME) mainly consists of various cell types and extracellular matrix, that communicate with cancer cells and promote cancer progression 5. Cancer-associated fibroblasts (CAFs), as the most common cells in TME 6, can lead to cancer progression via cancer stem-cell renewal, blunting immunotherapy efficacy, and chemoresistance 7, 8. However, the roles and mechanisms of immunosuppressive features of CAFs in OS deserve further investigation.

Lysyl oxidase (LOX) is a secreted enzyme in the extracellular matrix. Collagen and elastin as the substrates of LOX, are the main components of bone and lung, indicating that LOX may play a role in OS tumorigenesis and metastasis 9. Researchers have found that LOX is dysregulated in OS cell lines and confirmed its role in OS tumorigenesis and metastasis 10, 11. However, Xu et al. suggested that LOX acts as a tumor suppressor to inhibit the growth of OS cells 12. Therefore, the above contradictory outcomes indicate that the role of LOX is poorly understood in OS.

Single-cell RNA sequencing (scRNA-seq) is a powerful new tool to detect genetic and functional heterogeneity, reconstruct cellular components and their interactions, and detect rare subpopulations of TME 13, 14. ScRNA-seq is applied to identify and analyze the precise cellular clusters and compositions of OS 15, 16. However, all these studies had focused on cellular components, but had not clarified the roles and mechanisms of TME in OS progression clearly.

This study utilized scRNA-seq and bioinformatics technology to reveal the regulative roles of CAFs in TME of recurrent OS based on previous studies of GSE152048 and GSE162454. It might promote the understanding of the roles and mechanisms of CAFs in TME of recurrent OS at the single-cell level, which is deemed to provide novel therapeutic targets for OS.

## Materials and methods

### Single-cell RNA sequencing and bioinformatic analysis

The raw scRNA-seq data of GSE152048 and GSE162454 were downloaded and used for further bioinformatic analysis. We performed the data integration and the dimensionality reduction, cell-clustering and annotation, differentially expressed genes (DEGs) identification, and GO enrichment analysis, respectively.

### Pre-processing of scRNA-seq data

For each sample, the raw output data were processed in R software using the Seurat package (http://satijalab.org/seurat/) 17. We filtered out cells that expressed fewer than 300 genes or more than 10% mitochondrial genes of total expressed genes 18. The filtered cells were then utilized for the following bioinformatic analysis.

### Data integration and dimensionality reduction

The gene expression was calculated for each sample as the gene's fraction and multiplied by 10,000. The results were then converted to natural logarithms and normalized by adding 1 to prevent taking the log of 0. Before we carried out the principal component analysis (PCA) based on these highly variable genes (HVGs), we identified, centered, and scaled the top 3000 highly variable genes (HVGs) from the normalized expression matrix. On the basis of the top 50 PCA components found, the batch effects were eliminated by the R Harmony package 19.

### Patient samples

This study collected three paired primary and recurrent OS samples in our hospital. Each participant provided written informed consent for the data to be utilized in our study.

### Ethics committee approval and patient consent

This study was approved by the institutional review board and the medical ethics committee of Wuhan Union Hospital, Huazhong University of Science and Technology. Each participant provided written informed consent for the data to be utilized in our study. The animal study protocol was approved by the Committee on the Ethics of Animal Experiments of Huazhong University of Science and Technology.

### Western blot

The proteins were transferred to 0.22 μm polyvinylidene fluoride (PVDF) membranes (Merck Millipore, USA) and incubated with anti-N-cadherin, anti-E-cadherin, anti-LOX, and anti-GAPDH primary antibodies at 4 °C overnight. Secondary antibodies (Proteintech, Rosemont, IL, USA) were incubated with the following PVDF membranes before imaging.

### Cell migration assay

Cells were resuspended in 100 μl serum-free medium and were seeded on the upper chamber of the Transwell chamber plates (BD Biosciences, St Louis, MO, USA). The bottom well was used to store 20% FBS medium. After incubation at 37 °C for 24h, cells that migrated through the pores of the Transwell were fixed and stained.

### Wound healing assay

The treated tumor cells were seeded into 6-well plates to a final density of 1×10^5^ cells/well and incubated overnight to permit cell adhesion and formation of a confluent tumor cell monolayer. Wounds were generated by scratching lines with a 200 μl plastic tip in the cell monolayer. The wound closures were observed and recorded under a light microscope at 0h and 24h after the scratch was generated.

### Immunofluorescence (IF) staining

After high-temperature antigen retrieval, sections were incubated with blocking solution for 10 min at 25 °C, and the primary antibodies were applied overnight at 4 °C. The tissues were incubated with Polymer HRP-anti-mouse/Rabbit IgG secondary antibody. The sections were heat-treated after the application of each fluorophore and primary antibody, and then incubated with the secondary antibody and another fluorophore working solution. Tissue sections were counterstained with DAPI for 5 min.

### Immunohistochemistry (IHC) staining

All tissue sections were deparaffinized, rehydrated, and washed. Endogenous peroxidase was blocked using 3% hydrogen peroxide for 10min. After water-bath heating for antigen retrieval, slides were incubated with primary antibodies followed by HRP-linked secondary antibodies and diaminobenzidine staining. Counterstaining was done with hematoxylin. Slides were dehydrated with sequential ethanol washes.

### Subcutaneous xenograft tumor model

Nude mice (BALB/c, female, 4 to 5-week-old) were injected subcutaneously with 5×10^6^ MNNG/HOS cells. Tumor volumes were calculated with the length (a) and the width (b): volume (mm^3^) = ab^2^/2. The animals were sacrificed, and hematoxylin-eosin (HE) staining, IHC staining (Ki67, LOX, N-cadherin, and E-cadherin), IF staining (α-SMA, FAP, F4/80, CD163, iNOS, CD3, CD4, CD8), and TUNEL staining were performed to stain tumor tissues.

### TIMER database analysis

TIMER database investigates the immune infiltrates in various cancer types (https://cistrome.shinyapps.io/timer/). This study assessed the cumulative survival curves according to LOX expression in sarcoma tissues and the correlation of LOX with the abundance of immune infiltrates. Moreover, correlations between the expression of LOX and gene markers of epithelial-mesenchymal transition (EMT) (E-cadherin, N-cadherin, SNAI1, and S100A4) were explored via correlation modules.

### Statistical analysis

Statistical analyses were conducted by GraphPad 6.0 software. Two-tailed student's t-test was utilized to show a significant difference between two groups, and a one-way analysis of variance (ANOVA) was utilized for three or more groups. A p-value of < 0.05 was considered to indicate a statistically significant result (*p < 0.05 and **p < 0.01).

## Results

### Cellular constitution of OS tumor lesions

**Figure 1:**
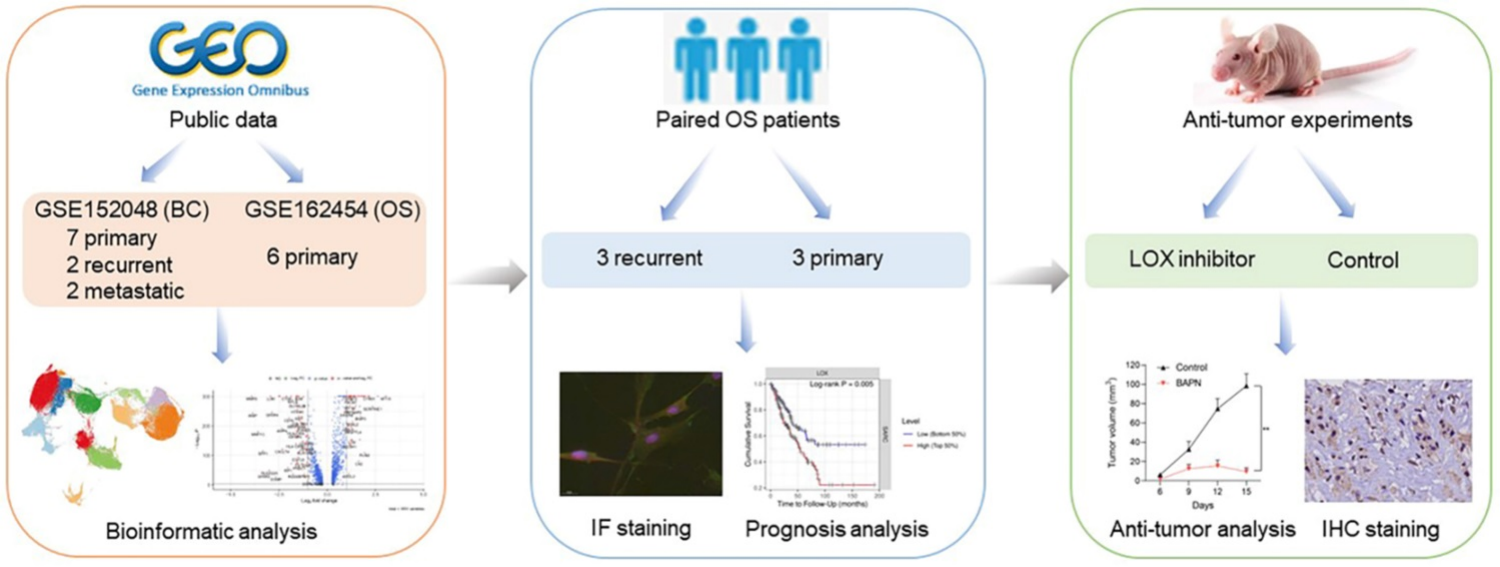
Graphical view of the study roadmap.

This study was designed and investigated as shown in Figure 1. We conducted scRNA-seq analyses to investigate the cellular constitution of primary, recurrent, and lung metastatic OS lesions according to the datasets of GSE152048 (BC) and GSE162454 (OS). GSE152048 (BC) includes 100,987 individual cells from 7 primary, 2 recurrent, and 2 lung metastatic OS lesions. GSE162454 (OS) includes 29,278 cells from six primary OS patients prior to neoadjuvant chemotherapy. Furthermore, we recruited three paired primary and recurrent OS samples to verify the results of scRNA-seq.

**Figure 2:**
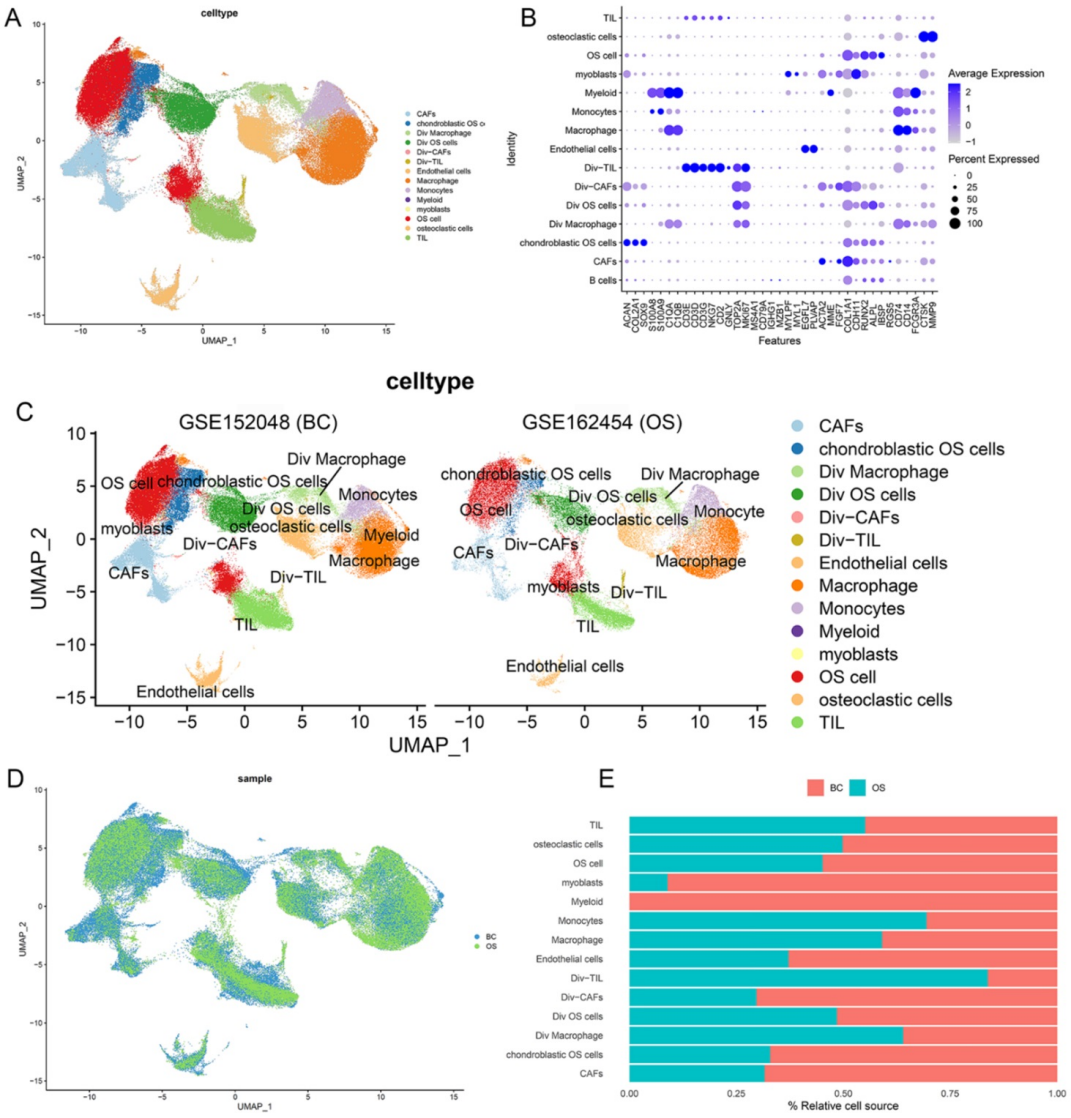
** Single-cell transcriptomic analysis of OS lesions. (A)** The t-SNE plot of 14 identified cell types in OS lesions. **(B)** Dot plots of the 37 signature gene expressions in 14 cellular clusters (log1p transformed). **(C, D)** The t-SNE plots of the 14 cell types in GSE152048 (BC) and GSE162454 (OS). **(E)** Relative proportion of each cell cluster in GSE152048 (BC) and GSE162454 (OS) was indicated.

After quality control, the cells of GSE152048 (BC) and GSE162454 (OS) were clustered into 14 major clusters via the T-distributed stochastic neighbor embedding (t-SNE) method (Figure 2A). Cluster-specific genes were utilized to annotate cell types with classic biomarkers. 37 signature gene expressions across the cellular clusters were shown by dot plots. The size of dots indicates the proportion of cells expressing the particular marker, and the spectrum of color represents the mean expressions of the markers (Figure 2B). Div-cells referred to the diverse cells with MKI67+ expression, which have enhanced proliferation ability. The t-SNE plots of the 14 cell types in GSE152048 (BC) and GSE162454 (OS) were shown (Figure 2C and 2D). The relative proportion of each cell cluster in GSE152048 (BC) and GSE162454 (OS) was indicated (Figure 2E).

### CAFs had a higher infiltrating level in recurrent OS

**Figure 3:**
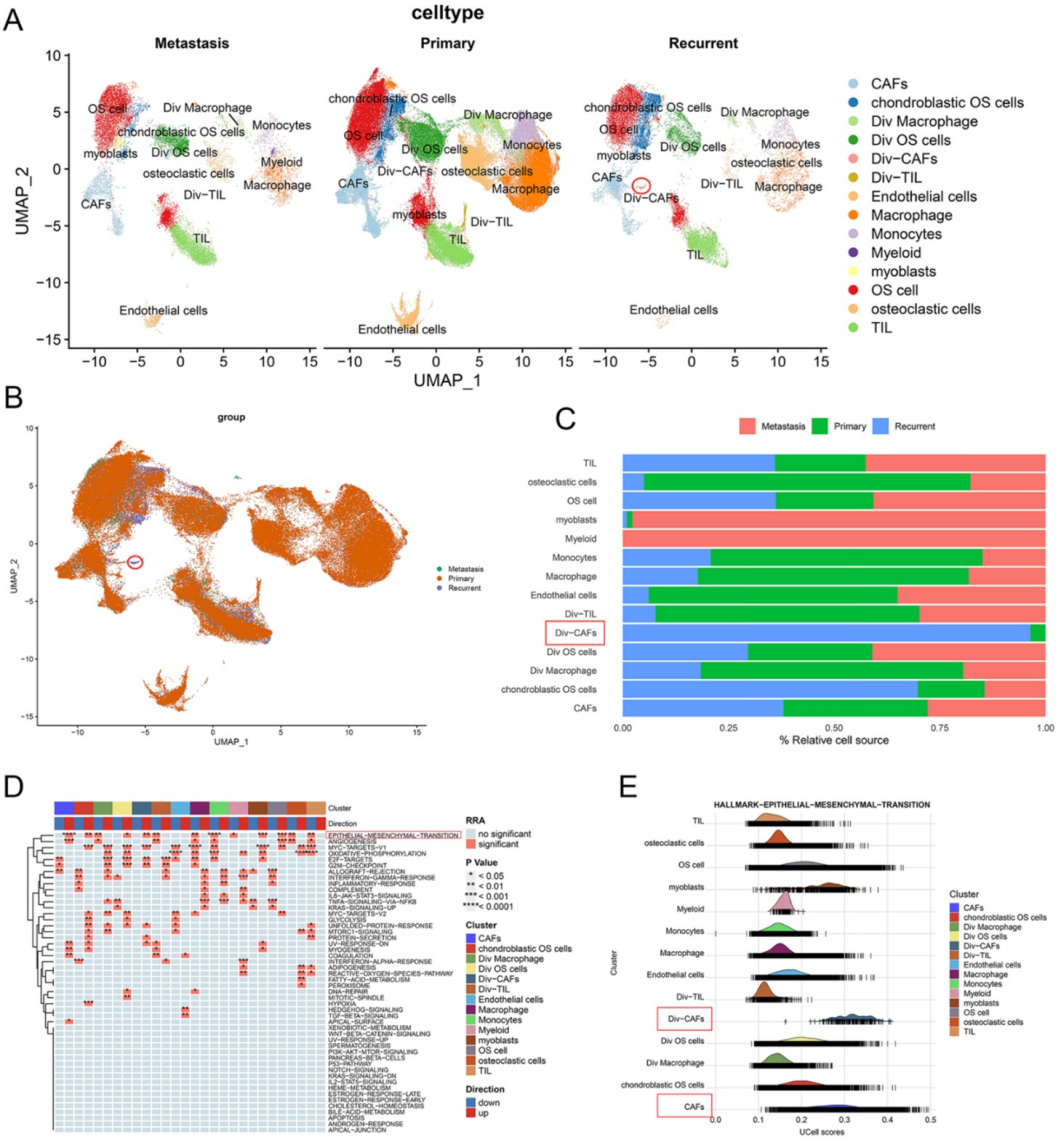
** CAFs had a higher infiltrating level in recurrent OS. (A, B)** The t-SNE plot of the 14 identified cell types in primary, recurrent, and lung metastatic OS lesions. **(C)** Relative proportion of each cell cluster across OS lesions was indicated. **(D)** The heatmap of GSEA of the 50 hallmark gene sets in the 14 cell clusters. **(E)** The ridgeplot of GSEA of EMT hallmark gene sets in the 14 cell clusters.

The t-SNE plot showed the 14 identified cell types in primary, recurrent, and lung metastatic OS lesions (Figure 3A and 3B). We further investigated the relative proportion of each cell cluster across primary, recurrent, and lung metastatic OS lesions. Remarkably, compared with primary samples, recurrent samples showed a significantly higher infiltrating level of Div-CAFs (96.42% vs 3.58%). No remarkable difference in CAFs was shown between primary and recurrent samples. Most of the identified cell types exist across patients. But the proportion of cell types varied among primary, recurrent, and lung metastatic samples because of the remarkable heterogeneity of TME (Figure 3C).

Moreover, this study investigated the DEGs in cells between recurrent and primary OS lesions. According to the pathway enrichment analysis via HALLMARK gene sets, the results showed the remarkably activated EMT pathway in recurrent OS tissues, especially in the cell cluster of CAFs (Figure 3D). The outcomes indicated the heterogeneity of expression patterns and activated pathways in cells between recurrent and primary OS. The ridgeplot of GSEA showed a significant correlation between EMT hallmark gene sets and the cell clusters of CAFs (Figure 3E). Accordingly, CAFs showed a higher infiltrating level and were enriched in the EMT pathway of recurrent OS.

### CAFs were correlated with EMT in recurrent OS patient tissues

**Figure 4:**
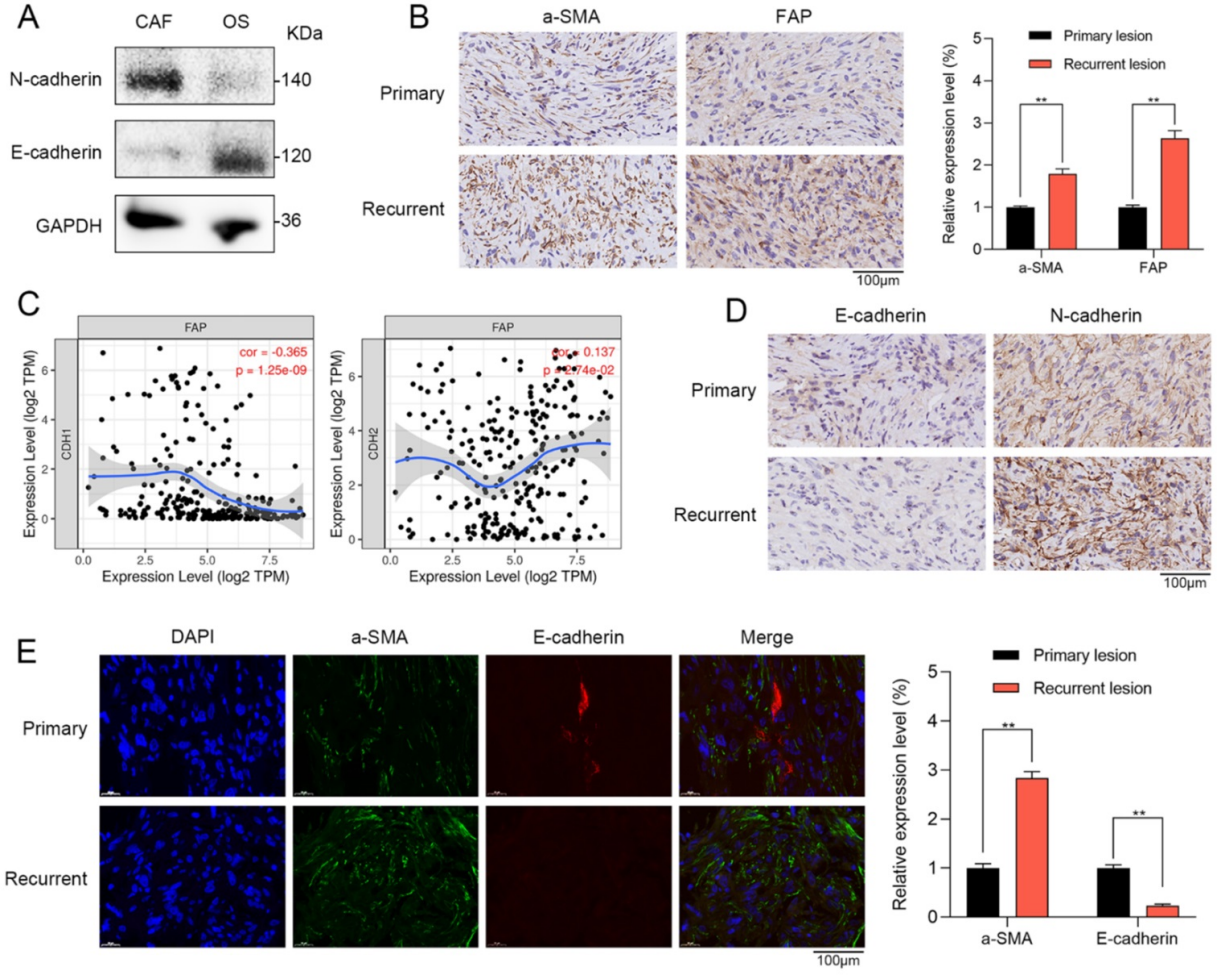
** CAFs were correlated with EMT in recurrent OS patient tissues. (A)** Western blot showed that CAFs had higher N-cadherin expression and lower E-cadherin expression when compared with OS cells. **(B)** The expressions of a-SMA and FAP were observed in primary and recurrent OS tissues via IHC staining (scare bar: 100 µm; **p < 0.01). **(C)** By TIMER database, FAP of CAFs has a significantly positive correlation with EMT markers of N-cadherin (r = 0.137, p = 2.74e-02) and negative association with E-cadherin (r = -0.365, p = 1.25e-09). **(D)** The expressions of E-cadherin and N-cadherin were observed in primary and recurrent OS tissues via IHC staining (scare bar: 100 µm). **(E)** Recurrent OS tissues were observed with higher a-SMA expression and lower E-cadherin expression when compared with primary OS tissues by IF staining (scare bar: 100 µm; **p < 0.01).

To further confirm the results of single-cell RNA sequencing and bioinformatic analysis, we investigated the role of CAFs in primary and recurrent OS patient tissues. Western blot showed that CAFs had higher N-cadherin expression and lower E-cadherin expression when compared with OS cells (Figure 4A). Furthermore, in the tissues of OS patients, we further verified that the stromal activation markers of CAFs such as recombinant stromal cell derived factor 1 (a-SMA) and fibroblast activation protein (FAP) were significantly higher in recurrent OS tissues when compared with primary OS tissues via IHC staining (Figure 4B). By TIMER database, FAP has a significantly positive correlation with EMT markers of N-cadherin (r = 0.137, p = 2.74e-02) and negative association with E-cadherin (r = -0.365, p = 1.25e-09) (Figure 4C). By IHC staining, recurrent OS tissues were observed with higher N-cadherin expression and lower E-cadherin expression when compared with primary OS tissues (Figure 4D). IF staining indicated that recurrent OS tissues had higher a-SMA expression and lower E-cadherin expression when compared with primary OS tissues (Figure 4E). Therefore, CAFs were significantly correlated with EMT markers in recurrent OS patient tissues.

### Expression pattern and clinical relevance of LOX expressed by CAFs

**Figure 5:**
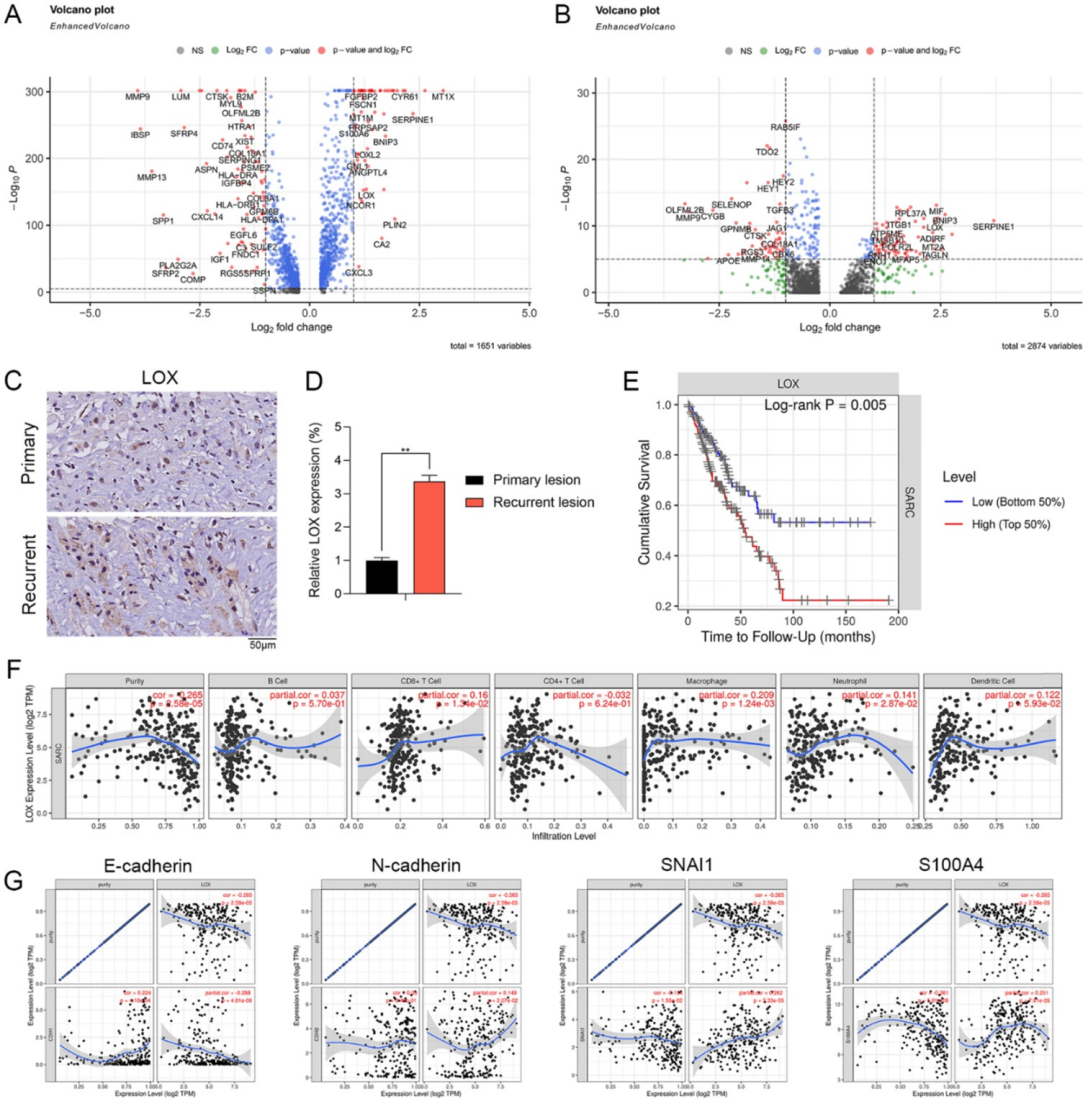
** Expression pattern and clinical relevance of LOX expressed by CAFs. (A)** The volcano plot indicates DEGs of CAFs between recurrent and primary OS. **(B)** The volcano plot indicates DEGs of Div-CAFs between recurrent and primary OS. **(C)** IHC staining showing the expression of LOX in paired recurrent and primary OS samples (scale bars: 50 µm). **(D)** qPCR showing the expression of LOX in paired recurrent and primary OS samples (**p < 0.01). **(E)** Cumulative survival curves according to LOX expression in sarcoma tissues via TIMER database. LOX high represented a higher LOX expression and showed a significantly poorer prognosis. **(F)** LOX has significant positive correlations with infiltrating levels of CD8+ T cells (r = 0.16, p = 1.34e-02) and macrophages (r = 0.209, p = 1.24e-03) in sarcoma via the TIMER database. **(G)** LOX has a significantly negative association with E-cadherin (r = -0.288, p = 4.81e-06) and positive correlations with N-cadherin (r = 0.148, p = 2.07e-02), SNAI1 (r = 0.262, p = 3.33e-05), and S100A4 (r = 0.251, p = 7.61e-05).

We further examined the DEGs in Div-CAFs and CAFs between recurrent and primary OS. The results showed that the expression level of LOX was remarkably higher in CAFs and Div-CAFs in recurrent OS (Figure 5A and 5B). IHC staining showing the expression of LOX was significantly higher in paired recurrent OS samples (Figure 5C). qPCR result also confirmed it (Figure 5D). Cumulative survival curves showed that a higher LOX expression was correlated with a significantly poorer prognosis of sarcoma via TIMER database (Figure 5E). LOX has significant positive correlations with infiltrating levels of CD8+ T cells (r = 0.16, p = 1.34e-02) and macrophages (r = 0.209, p = 1.24e-03) in sarcoma via the TIMER database (Figure 5F). LOX has a significantly negative association with E-cadherin (r = -0.288, p = 4.81e-06) and positive correlations with N-cadherin (r = 0.148, p = 2.07e-02), SNAI1 (r = 0.262, p = 3.33e-05), and S100A4 (r = 0.251, p = 7.61e-05) (Figure 5G). Therefore, LOX was associated with immune infiltration levels and EMT states of sarcoma, which may be a new target for OS treatment.

### LOX promoted recurrent OS *in vitro*

**Figure 6:**
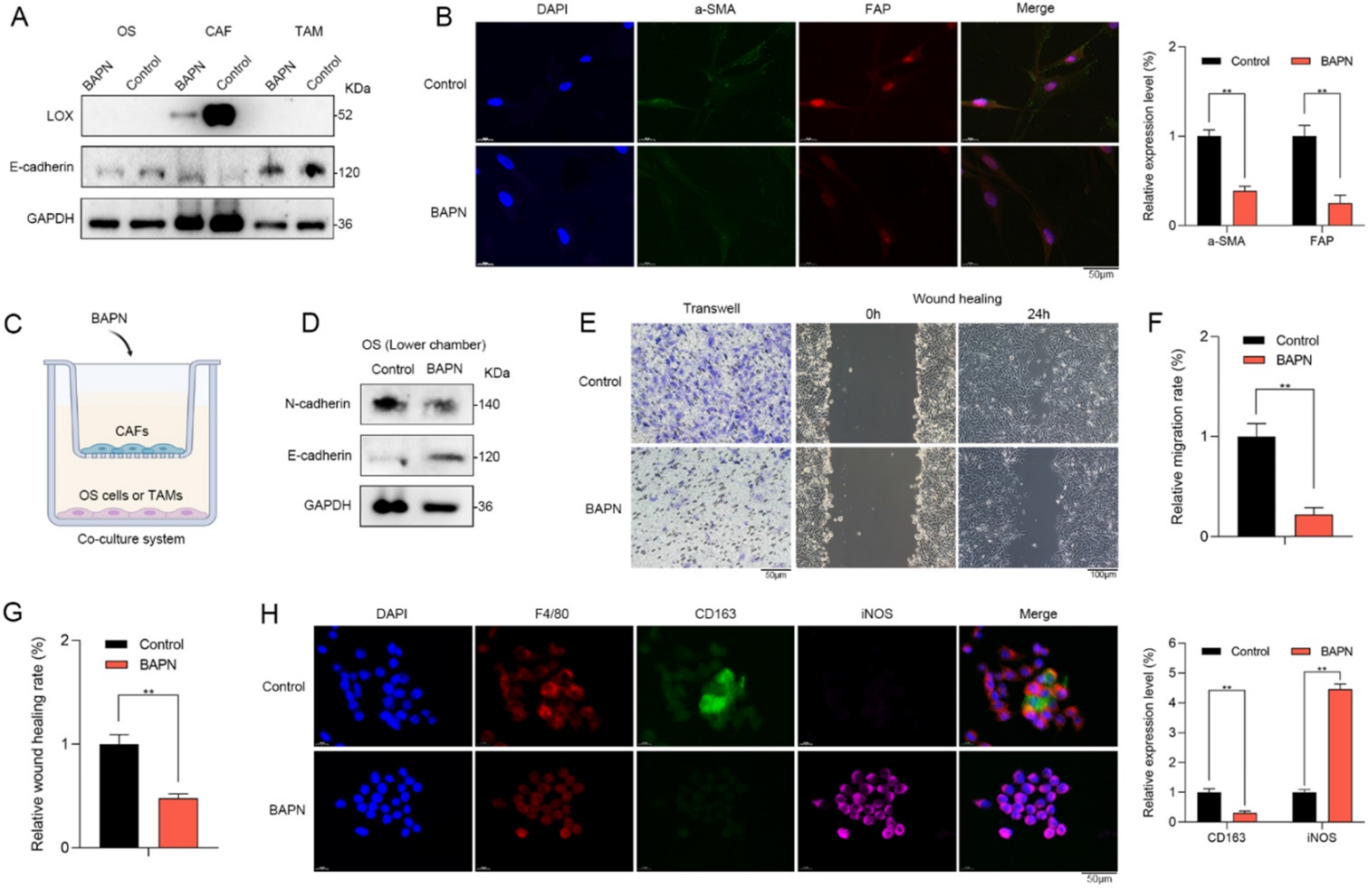
** LOX promoted recurrent OS *in vitro*. (A)** Western blot showed that LOX inhibition by BAPN could significantly downregulate the LOX expression and contribute to the expressional changes in EMT markers such as E-cadherin in CAFs. No changes in LOX expression of OS cells or macrophages were observed. **(B)** CAFs in BAPN group had lower expression levels of a-SMA and FAP when compared with control group (scale bars: 50 µm; **p < 0.01). **(C)** To explore the roles of LOX in the TME, we utilized the co-culture system. **(D)** In the lower chamber, western blot showed that BAPN group had lower N-cadherin expression and higher E-cadherin expression when compared with control group. The abilities of **(E, F)** migration (scale bars: 50 µm; **p < 0.01) and **(E, G)** wound healing (scale bars: 100 µm) of OS cells were significantly inhibited in BAPN group. **(H)** In BAPN group, the relative proportion of M2 macrophages (CD163) was significantly decreased (scale bars: 50 µm; **p < 0.01).

β-aminopropionitrile (BAPN) is a specific irreversible LOX inhibitor, that targets the active sites of LOX or LOXL isozymes 20. Western blot showed that LOX inhibition by BAPN could significantly downregulate the LOX expression in CAFs. However, no changes in LOX expression of OS cells or macrophages were observed (Figure 6A). Therefore, LOX inhibition might have no direct effects on OS cells or macrophages. Furthermore, LOX inhibition contributed to the expressional changes in EMT markers such as E-cadherin (Figure 6A) as well as stromal activation markers of CAFs such as a-SMA and FAP *in vitro* (Figure 6B). Therefore, LOX might play a vital role in regulating the function of CAFs.

To further explore the regulative roles of LOX in the TME of OS, we utilized the co-culture system (Figure 6C). In the upper chamber, CAFs were treated with BAPN. In the lower chamber, we found that OS cells of BAPN group had lower N-cadherin expression and higher E-cadherin expression when compared with control group (Figure 6D). Moreover, the abilities of migration and wound healing of OS cells were significantly inhibited in BAPN group (Figure 6E-G). In BAPN group, the relative proportion of M2 macrophages (CD163) was significantly decreased (Figure 6H). Accordingly, LOX might promote recurrent OS via regulating the function of CAFs and macrophage polarization.

### The anti-OS effects of LOX inhibition *in vivo*

**Figure 7:**
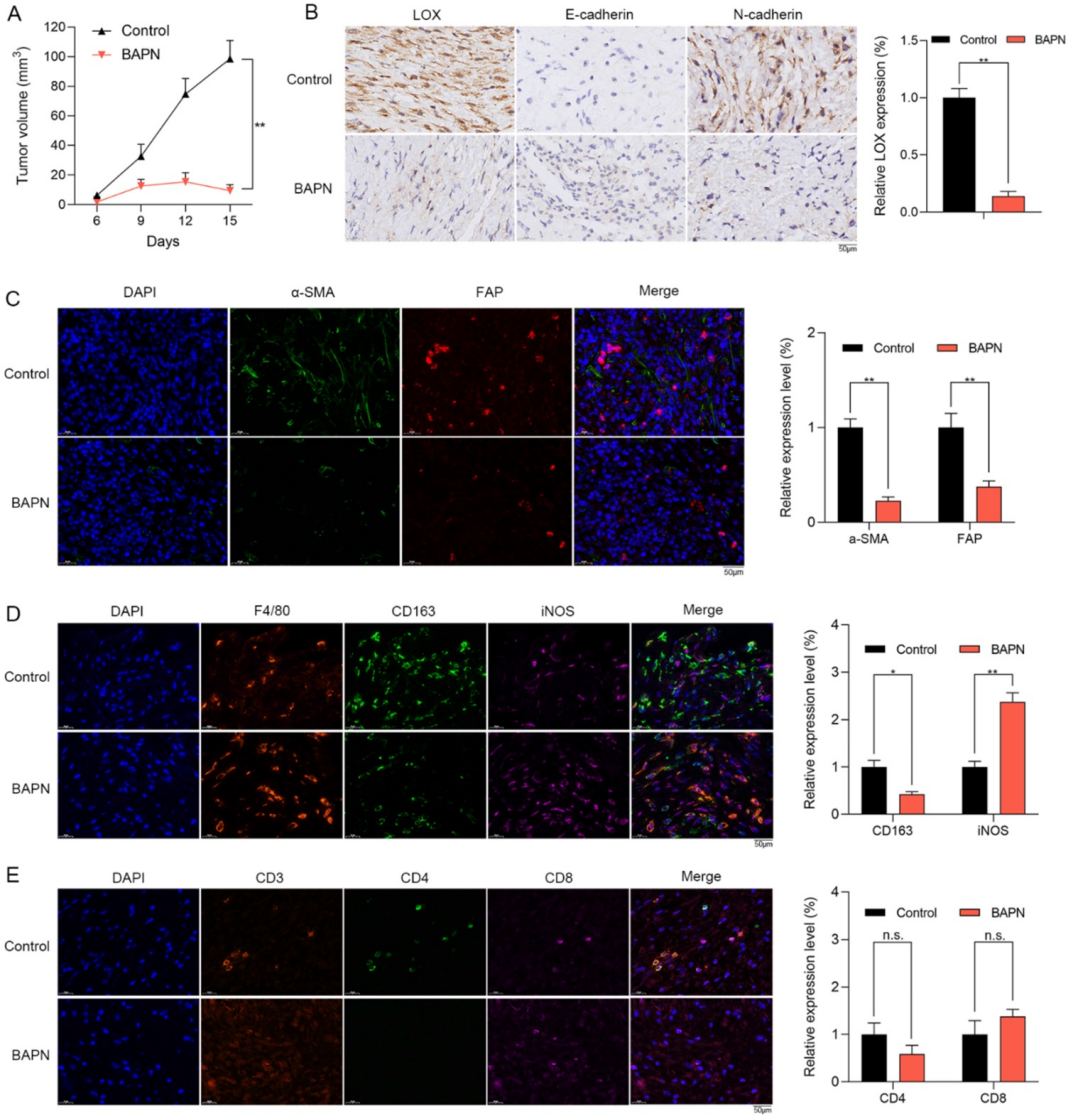
** The anti-OS effects of LOX inhibitor *in vivo*. (A)** Mice were divided into control and BAPN groups. The volumes of subcutaneously transplanted tumors were detected (**p < 0.01). **(B)** IHC staining of LOX, N-cadherin, and E-cadherin in tumors (scare bar: 50 µm; **p < 0.01). **(C)** Relative proportion of CAFs in tissues were shown by IF staining (scale bars: 50 µm; **p < 0.01). **(D)** Relative proportion of macrophages in tissues were shown by IF staining (CD163: M2 type of macrophages; iNOS: M1 type of macrophages; scale bars: 50 µm; **p < 0.01, *p < 0.05). **(E)** Relative proportion of T cells in tissues were shown by IF staining (scale bars: 50 µm; n.s.: no significance).

The effects of LOX inhibition on subcutaneous xenograft tumor model were explored. Mice were treated with PBS (control group), and BAPN (BAPN group) for 15 days. Compared with control group, BAPN significantly decreased the tumor volume (Figure 7A). IHC staining showed that the expression of LOX was significantly decreased in BAPN group. Furthermore, the lower N-cadherin expression and higher E-cadherin were observed in BAPN group (Figure 7B). The above results suggested that BAPN inhibits the LOX expression and has anti-OS effects *in vivo*.

In the subcutaneous xenograft tumor model, we found that the stromal activation markers of CAFs such as a-SMA and FAP in tissues were significantly lower in BAPN group via IF staining (Figure 7C). In BAPN group, the relative proportions of M2 macrophages (CD163) were significantly decreased (Figure 7D) and the infiltrating levels of CD8+ T cells were decreased with no significant change (Figure 7E). Therefore, LOX inhibition might regulate macrophage polarization and remodel the TME of OS *in vivo*.

## Discussion

As for recurrent tumors, patients often fail to get surgical resection and pathological specimens. Therefore, recurrent tumors are mainly treated according to the pathological features of primary tumors. However, the great differences in TME between primary and recurrent tumors have been uncovered 21. Taking the high heterogeneity and poor prognosis of recurrent OS into account, it is of great importance to reveal the pathological features of recurrent OS as precisely as possible.

With the development of scRNA-seq technology, it holds great promise for identifying and analyzing the precise cellular clusters and compositions in TME of OS. This study indicated a high CAFs infiltration level in recurrent OS. CAFs as the main component of TME, contribute to cancer progression 8. CAFs might secrete IL-6 and remodel the immunosuppressive TME in esophageal cancer 22. Previous researches have indicated that CAFs in TME might reduce the immunotherapy response in many cancers 6. Studies revealed that CAFs of OS formed three distinct subclusters with novel gene markers and functions 15, 16. In this study, recurrent OS was characterized by abundant CAFs infiltration and CAFs were enriched in the EMT pathway. Therefore, it provided evidence of OS recurrence induced by CAFs.

**Figure 8:**
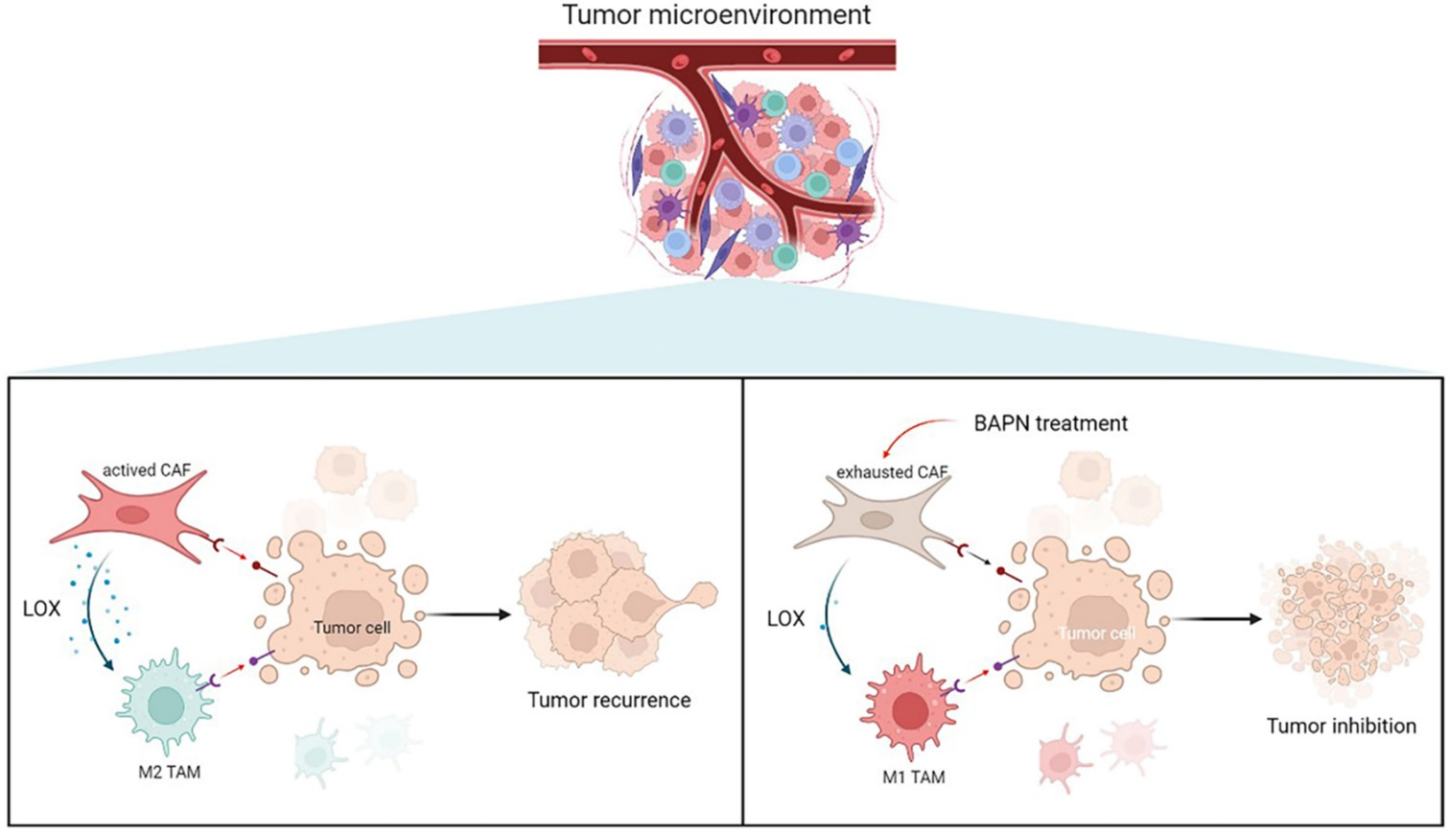
Overview of targeting LOX of CAFs to remodel TME and treat recurrent OS.

To further investigate the underlying mechanisms of CAFs in EMT and recurrence of OS, the gene expression pattern of CAFs was detected. This study showed that LOX had the highest expression level in CAFs among cell types in recurrent OS. Emerging studies have shown that LOX upregulation promotes the progression and metastasis of different tumors 23. LOX functions in VEGF induction, HIF-1α activation, and promoting angiogenesis and EMT process in various tumors, which underlines the role of LOX-1 as a potential therapeutic target for current antitumor strategies 24. Our study confirmed that LOX might contribute to poor prognosis in sarcoma patients. LOX was significantly associated with CD8+ T cells and macrophages infiltration, and EMT states of sarcoma. In this study, LOX inhibition by BAPN could regulate CAFs function and induce macrophage polarization, thereby further inhibiting OS (Figure 8).

In spite of the novel findings in our study, we have to clarify some limitations and further studies are warranted to improve them. The scRNA-seq data of OS tissues from two public datasets were limited and unpaired. We only confirmed the outcomes in three paired primary and recurrent OS samples, the outcomes of this study should be further validated with larger paired samples. We utilized the LOX inhibitor to investigate the role of LOX in OS both *in vivo* and *in vitro*. More underlying mechanisms and clinical trials deserve further investigation to bring novel insights into the treatment of recurrent OS.

## Conclusions

In summary, this study investigated the heterogeneity of recurrent OS and highlighted an innovative mechanism that CAFs regulate EMT of OS via LOX. Targeting LOX of CAFs showed promising efficacy in remodeling TME and treating recurrent OS. Our study indicates a new mechanism of CAFs in regulating tumor immune microenvironment and sheds light on a unique approach to improving the prognosis of recurrent OS.

## Supplementary Material

Supplementary material.
